# Elucidating Role of Reactive Oxygen Species (ROS) in Cisplatin Chemotherapy: A Focus on Molecular Pathways and Possible Therapeutic Strategies

**DOI:** 10.3390/molecules26082382

**Published:** 2021-04-19

**Authors:** Sepideh Mirzaei, Kiavash Hushmandi, Amirhossein Zabolian, Hossein Saleki, Seyed Mohammad Reza Torabi, Adnan Ranjbar, SeyedHesam SeyedSaleh, Seyed Omid Sharifzadeh, Haroon Khan, Milad Ashrafizadeh, Ali Zarrabi, Kwang-seok Ahn

**Affiliations:** 1 Department of Biology, Faculty of Science, Islamic Azad University, Science and Research Branch, Tehran 1477893855, Iran; sepidehmirzaei.smv@gmail.com; 2 Department of Food Hygiene and Quality Control, Division of Epidemiology, Faculty of Veterinary Medicine, University of Tehran, Tehran 1417466191, Iran; houshmandi.kia7@ut.ac.ir; 3 Young Researchers and Elite Club, Tehran Medical Sciences, Islamic Azad University, Tehran 1477893855, Iran; ah_zabolian@student.iautmu.ac.ir (A.Z.); h.saleki@student.iautmu.ac.ir (H.S.); smohammad77.tr@gmail.com (S.M.R.T.); adnan.ranjbar98@gmail.com (A.R.); Somid.sharifzadeh@gmail.com (S.O.S.); 4 Student Research Committee, Iran University of Medical Sciences, Tehran 1449614535, Iran; Hesammedical982@gmail.com; 5 Department of Pharmacy, Abdul Wali Khan University, Mardan 23200, Pakistan; hkdr2006@gmail.com; 6 Faculty of Engineering and Natural Sciences, Sabanci University, Orta Mahalle, Üniversite Caddesi No. 27, Orhanlı, Tuzla, Istanbul 34956, Turkey; milad.ashrafizadeh@sabanciuniv.edu; 7 Sabanci University Nanotechnology Research and Application Center (SUNUM), Tuzla, Istanbul 34956, Turkey; alizarrabi@sabanciuniv.edu; 8 Department of Science in Korean Medicine, College of Korean Medicine, Kyung Hee University, Seoul 02447, Korea

**Keywords:** cisplatin, reactive oxygen species, drug resistance, chemoresistance, nanoparticles, gene therapy, anti-cancer therapy

## Abstract

The failure of chemotherapy is a major challenge nowadays, and in order to ensure effective treatment of cancer patients, it is of great importance to reveal the molecular pathways and mechanisms involved in chemoresistance. Cisplatin (CP) is a platinum-containing drug with anti-tumor activity against different cancers in both pre-clinical and clinical studies. However, drug resistance has restricted its potential in the treatment of cancer patients. CP can promote levels of free radicals, particularly reactive oxygen species (ROS) to induce cell death. Due to the double-edged sword role of ROS in cancer as a pro-survival or pro-death mechanism, ROS can result in CP resistance. In the present review, association of ROS with CP sensitivity/resistance is discussed, and in particular, how molecular pathways, both upstream and downstream targets, can affect the response of cancer cells to CP chemotherapy. Furthermore, anti-tumor compounds, such as curcumin, emodin, chloroquine that regulate ROS and related molecular pathways in increasing CP sensitivity are described. Nanoparticles can provide co-delivery of CP with anti-tumor agents and by mediating photodynamic therapy, and induce ROS overgeneration to trigger CP sensitivity. Genetic tools, such as small interfering RNA (siRNA) can down-regulate molecular pathways such as HIF-1α and Nrf2 to promote ROS levels, leading to CP sensitivity. Considering the relationship between ROS and CP chemotherapy, and translating these findings to clinic can pave the way for effective treatment of cancer patients.

## 1. Introduction

The field of cancer chemotherapy is suffering from a number of challenges; drug resistance is the most significant. In respect to the benefits of chemotherapy in the treatment of cancer patients, factors responsible for mediating chemoresistance should be identified in further studies, in order to prevent drug resistance [1,2,3,4,5,6,7]. Cisplatin (CP) is a platinum-containing drug that was first discovered in 1965 and became famous due to its great antimicrobial activity. More experiments demonstrated that platinum-containing agents can possess anti-cancer activity [8,9,10,11,12,13]. As an electrophilic reagent, platinum can interact with nucleophilic residues of nucleobases, including guanine and adenosine by forming covalent bonds. Due to the presence of nucleophilic residues on a wide variety of cellular components, platinum-containing compounds can interact with ribosomes, spliceosomes, RNA and proteins [14,15,16,17]. The major pathway for suppressing cancer progression by CP is inducing DNA damage by forming adducts with DNA, resulting in apoptosis and cell cycle arrest [18]. More efforts in revealing anti-tumor activity of CP revealed that CP has the capacity of internalization in organelles, such as endoplasmic reticulum (ER), mitochondrion, lysosomes, and nucleus. This demonstrates that, in addition to DNA damage, CP can induce cell death by impairing homeostasis of vital organelles, such as ER and mitochondrion [19,20]. However, this impact may negatively affect anti-tumor activity of CP. It has been reported that in spite of impairing homeostasis of proteins and organelles in cytoplasm upon CP accumulation, pro-survival mechanisms, such as autophagy, unfolded protein response (UPR) and other protective processes may be activated [21,22,23]. These mechanisms may induce cancer cells resistance to CP chemotherapy.

Upon administration, CP immediately emerges in blood circulation. A high amount of CP (up to 98%) can be found in status of connected to plasma proteins, such as human serum albumin (HAS) [24,25]. Each HAS can bind to five CP molecules. One of the problems in patients receiving CP is the emergence of zinc imbalance. This is due to binding capacity of HAS-CP to histidine residues that are involved in transportation of Zn^2+^ ions in cells [26,27]. The penetration of CP into cells is performed via passive diffusion [28].

The benefits of using CP in cancer chemotherapy became absent as a result of chemoresistance. Cancer cells no longer become responsive to CP chemotherapy and can upregulate molecular pathways to induce drug resistance [29,30,31]. A wide variety of factors are considered as key players in mediating CP resistance. Drug transporters participate in triggering CP resistance. ATP7A and ATP7B are copper transporters that can bind to cysteine residue of CP to diminish its internalization in cells, leading to chemoresistance [32]. It has been reported that enhanced activity and expression of P-glycoprotein (P-gp) can also stimulate CP resistance [33]. On the other hand, in CP-resistant cancer cells, pro-apoptotic factors, such as BCL2 associated X (BAX) undergo down-regulation, while an increase occurs in the expression of anti-apoptotic factors, such as Bcl-2 to trigger CP resistance [34,35]. It seems that glutathione peroxidase 4 (GPX4) upregulation prevents ferroptosis in cancer cells to mediate CP resistance [31]. In this case, the inhibition of these antioxidant agents can predispose cancer cells to CP chemotherapy. In head and neck cancer cells, down-regulating glutaredoxin 5 stimulates ferroptosis, leading to CP sensitivity [36]. Transcriptional activation of RAD51 by CtBP1 results in CP resistance [37]. Noteworthy, it appears that CP administration can significantly promote metastasis and invasion of cancer cells by inducing macrophages [38]. The experiments have also tried to target molecular pathways involved in CP resistance via anti-tumor agents. For instance, propofol and hederagenin are among anti-tumor agents that can promote CP sensitivity of cancer cells by down-regulating Wnt signaling and suppressing autophagy [2,39].

As mentioned earlier, the impact of CP on intracellular organelles might pave the way for CP resistance. In the present review, our aim is to reveal the role of reactive oxygen species (ROS) in mediating/suppressing CP resistance. This review focuses on molecular pathways to relate ROS generation with efficacy of CP chemotherapy in cancer therapy. Future experiments can focus on targeting molecular pathways involved in this review articles and we have provided some examples in this case.

## 2. ROS: Dual Role in Cancer Progression/Inhibition

### 2.1. Basics

Reactive species have gained much attention in the field of biology and medicine, and to date, different kinds of reactive species have been recognized, based on their source, being either oxygen, nitrogen or sulfur [40,41,42]. ROS are derived from oxygen through some reactions such as reduction-oxidation reactions or electronic excitation [43]. There are four major types of ROS, including superoxide, hydrogen peroxide, peroxyl radical and lipid peroxidase [44,45,46]. As chemically active free radicals, ROS play a remarkable role in tissue homeostasis. The production of ROS occurs in mitochondrion and this is performed during mitochondrial respiration and inducing the partial reduction of oxygen [45,47,48]. In addition to mitochondria, other cellular organelles, such as ER and peroxisomes can participate in ROS formation [49,50]. It has been reported that ROS can interact with proteins, lipids and genetic materials in cells [51,52]. The imbalance in the generation of ROS can lead to the emergence of oxidative stress with the dual role of being beneficial or harmful. The physiological functions of cells, such as aging, inflammation and immune responses are governed by ROS [53,54,55]. Therefore, the presence of ROS is vital for normal function of cells. However, increased levels of ROS production can result in the development of pathological events, including neurodegenerative diseases, diabetes and cancer [56,57,58].

ROS participate in redox signaling and in this case, their low level generated by mitochondrial respiration or nicotinamide adenine dinucleotide phosphate oxidase (NADPH) oxidase (NOX) is required [59]. In redox signaling, ROS regulate a variety of molecules, including protein kinases and transcription factors to monitor proliferation, differentiation, migration and cytokine production. The opposite term of redox signaling is redox modulation that ROS action does not rely on first messenger (extracellular stimuli) and ROS induce changes in characteristics of redox-sensitive molecules, such as nucleic acid and metabolic enzymes [60]. One of the most well-known pathways that ROS participate is apoptosis induction. Enhanced generation of ROS disrupts mitochondrial homeostasis, and this leads to the upregulation of apoptotic factors, such as Bax and Bid, and down-regulation of anti-apoptotic factors, such as Bcl-2. Then, the release of cytochrome C (cyt C) from mitochondrion occur, leading to activation of caspase cascade and apoptotic cell death. Furthermore, ROS can impair ER homeostasis to stimulate apoptosis [61].

### 2.2. ROS Role in Cancer

In the previous section, we have summarized the role of ROS production, their role in physiological conditions and related pathways. In this section, an overview of the ROS role in cancer progression/inhibition is provided to shed some light on its targeting pathways in cancer therapy. The molecular pathways that are regulated by ROS are of importance in cancer therapy [62,63,64]. Increased ROS generation leads to the activation of p38 and extracellular signal-regulated kinase (ERK), which subsequently stimulates cell death and cell cycle arrest at S and G2/M phases [65]. Organelles are vital targets of ROS in cancer cells. Upon ROS overgeneration, ER stress occurs, and related molecular pathways, including glucose regulated protein 78 (GRP78) and C/EBP homologous protein (CHOP) undergo upregulation that trigger anti-tumor activity [66]. Previously, it was mentioned that an increase in ROS generation impairs mitochondrial homeostasis. It has been reported that by triggering mitochondrial damage, ROS promotes the expression level of FOXO3a in mediating its nuclear translocation. In the nucleus, FOXO3a enhances expression level of tumor-suppressing factors, such as Bim caspase-3 and phosphatase and tensin homolog (PTEN) to induce apoptosis in cancer cells [67]. This study clearly demonstrates that by regulating mitochondria, ROS can induce apoptosis. In addition to apoptotic cell death, ROS overgeneration can stimulate ferroptosis in decreasing proliferation and viability of cancer cells [68]. Autophagy is another programmed cell death (PCD) mechanism that can be stimulated by ROS levels in cancer therapy [69,70]. In lung cancer cells, increased ROS generation leads to stimulation of mitogen-activated protein kinase (MAPK) that in turn, induces ERK and c-Jun N-terminal kinase (JNK) pathways. Then, autophagic cell death occurs that remarkably diminishes proliferation and growth of lung cancer cells [71]. Therefore, elevating ROS generation is the most important pathway that anti-tumor agents follow in cancer elimination [72]. One of the forms of autophagy is mitophagy that degrades damaged mitochondrion [73]. ROS overgeneration leads to mitochondrial injury and provides the conditions for mitophagy, resulting in a decrease in cancer cell viability [74]. Noteably, in respect to the role of ROS in reducing cancer cell viability, it has been reported that cancer stem cells (CSCs) preserve ROS generation at low levels to obtain chemoresistance [75]. Therefore, using agents that enhance ROS generation is improtant in providing chemosensitivity. Overall, studies are in agreement with anti-tumor activity of ROS and their capacity in regulating various molecular pathways [76,77,78,79,80]. However, there are controversies about the role of ROS in cancer cells. Although previous statements demonstrate the role of ROS as anti-tumor agents, there are experiments showing the tumorigenesis role of ROS. Immune system plays a significant role in cancer therapy. In impairing anti-tumor activity of immune system, cancer-associated fibroblasts (CAFs) enhance ROS generation to provide polarization of monocytes to myeloid-derived suppressor cell (MDSC) [81]. It appears that hepatitis B virus (HBV) can enhance ROS generation in hepatocellular carcinoma. Enhanced ROS production leads to IQGAP1 and Rac1 interaction that overexpressed Rac1 induces Src/FAK signaling via phosphorylation to promote migration and invasion of cancer cells, and stimulate anoikis resistance [82]. These studies demonstrate the dual role of ROS in cancer. In the next sections, a mechanistic discussion of ROS role in CP sensitivity/resistance is provided [83].

## 3. ROS, Cisplatin Chemotherapy and Related Molecular Pathways

### Cisplatin Sensitivity

The Krüppel-like factor 4 (KLF4) is a zinc finger-containing transcription factor capable of regulating different biological activities such as differentiation and tumorigenesis. The interaction partner and cell type determine role of KLF4 as a tumor-suppressing or tumor-promoting factor [84]. The overexpression of KLF4 is in favor of enhancing CP-mediated apoptosis in cancer cells [85]. In CP resistant-cancer cells, KLF4 and ROS undergo down-regulation that are responsible for increased cell viability [86]. As their levels decrease simultaneously, KLF4 upregulation may promote ROS levels in enhancing CP sensitivity of cancer cells.

MicroRNAs (miRNAs) are regulators of different biological processes in cells, such as proliferation, migration, differentiation, apoptosis and autophagy [87]. In addition to physiological roles, miRNAs also play a significant role in pathological events via regulating various molecular pathways [88]. MiRNA-124 is a new emerging miRNA in cancer chemotherapy that its upregulation down-regulates oncogenic signal transducer and activator of transcription 3 (STAT3) pathway to promote CP sensitivity [89]. Furthermore, it can be considered as a biomarker for determining response to CP chemotherapy, so that gastric cancer patients with low levels of miRNA-124 have poor response to CP chemotherapy [90]. Noteworthy, miRNA-124 can regulate ROS levels in affecting CP response of cancer cells. In this way, miRNA-124 decreases SIRT1 expression to increase ROS levels that subsequently, stimulate JNK phosphorylation, leading to increased CP sensitivity of hepatocellular carcinoma cells [91]. The same phenomenon occurs by miRNA-519d in colorectal cancer cells. MiRNA-519d is a critical regulator of cancer response to CP chemotherapy. MiRNA-519d can reduce expression level of XIAP to potentiate CP cytotoxicity against cancer cells [92]. Furthermore, miRNA-519d impedes CP resistance by inducing apoptosis through MCL-1-dependent mitochondrial pathway [93]. In colorectal cancer cells, miRNA-519d down-regulates the expression level of tripartite motif 32 (TRIM32) to enhance ROS levels, leading to mitochondrial dysfunction and increased CP sensitivity [94]. Investigating the expression level demonstrates that miRNAs with tumor-suppressing role undergo down-regulation in CP resistant-cancer cells. Such phenomenon is obvious in cervical cancer in which miRNA-497 shows low expression, while an increase occurs in expression profile of transketolase (TKT) (upregulation in 81.1% of samples). By reducing TKT expression, miRNA-497 promotes ROS levels, while induces GSH depletion, leading to cancer cell death and CP sensitivity [95].

Recent experiments have focused on revealing role of sirtuin-2 (SIRT2) in cancer and providing rationale for its therapeutic targeting [96]. SIRT2 can suppress migration and invasion of cancer cells via isocitrate dehydrogenase 1 (IDH1) deacetylation [97]. Furthermore, SIRT2 can inhibit proliferation and colony-formation capacity of cancer cells [98]. In ovarian cancer cells, enhancing SIRT2 expression paves the way for CP sensitivity. CP administration significantly increases ROS levels to induce SIRT2 expression, resulting in ovarian cancer suppression [78].

**Figure 1:**
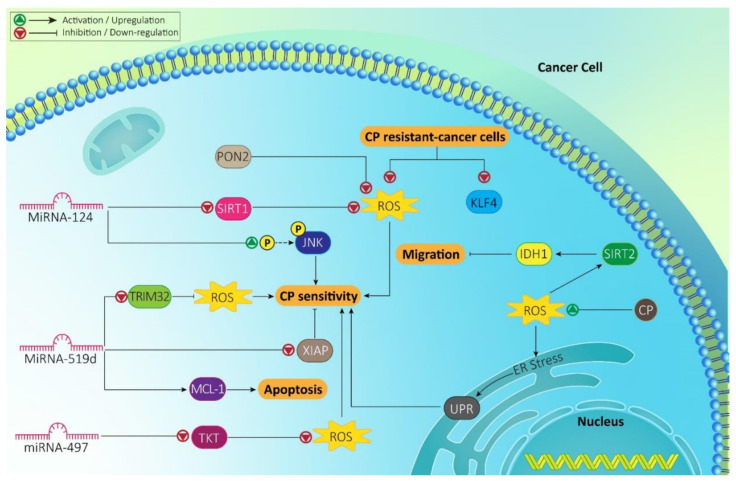
Molecular pathways regulating ROS generation and their role in CP sensitivity. The interesting point is the overgeneration and inhibition of ROS levels in CP sensitivity. ROS can affect migration and proliferation of cancer cells in CP sensitivity. MiRNAs can also function as upstream mediators of ROS in CP sensitivity.

One of the targets in cancer therapy is ER, so that inducing ER stress enhances efficacy of chemotherapy in cancer eradication [99]. Triggering ER stress and activating UPR are followed by CP in cancer treatment [100]. In ovarian cancer cells, CP enhances ROS levels to induce ER stress. Then, UPR activates that overcomes drug resistance [101]. It seems that ROS levels can be considered as a biomarker for predicting response of cancer cells to chemotherapy. For this purpose, Sun and colleagues have developed a scoring system, based on ROS, for predicting cancer patients’ response to CP chemotherapy. In this system, there are 25 scores in which scores 0–12 demonstrate low score groups, while scores 13–25 show high score groups. As ROS overgeneration enhances CP sensitivity and apoptosis induction, by enhancing ROS levels, patients are included in high score groups, which have high overall survival and good prognosis [102]. This score can be used in clinical course. Furthermore, down-regulating molecular pathways modulating ROS can pave the way for CP sensitivity. The human paraoxonase (PON) family has three distinct members including PON1, PON2 and PON3. PON1 and PON3 are expressed in the liver, while PON2 demonstrates expression in various tissues and intracellular accumulation upon translation [103]. It has been reported that PON2 possesses antioxidant activity in different tissues, such as the intestine and nervous system [104,105,106]. The overexpression of PON2 is correlated with CP resistance. In order to increase CP sensitivity of melanoma cells, silencing PON2 promotes ROS levels, resulting in decreased viability and proliferation [107]. Figure 1 and Table 1 demonstrate an overview of molecular pathways involved in CP sensitivity via ROS regulation.

## 4. Cisplatin Resistance

Inhibiting the expression of molecular pathways that reduce ROS levels and confer CP resistance is important in effective cancer chemotherapy. That is the reason why experiments have focused on the identification of such pathways and disrupting their expression. In head and neck cancers, ROS inhibition is associated with CP resistance. Enhancing ROS levels mediates ferroptosis and cell death. Nuclear factor erythroid 2-related factor 2 (Nrf2) is suggested to diminish ROS levels upon CP chemotherapy of head and neck cancer cells. Nrf2 signaling inhibition promotes ROS levels, potentiating ferroptosis and providing CP sensitivity [115].

It seems that ROS can provide metabolic reprograming to enhance resistance of non-small cell lung cancer (NSCLC) cells to CP. In this way, exposing NSCLC cells to CP is associated with an increase in mitochondrial function, PPAR-gamma coactivator-1α (PGC-1α) and mitochondrial. Simultaneously, glycolysis down-regulation occurs, but this does not affect cell cycle progression of cancer cells. These metabolic changes are mediated via ROS, so that ROS can promote PGC-1α expression and mitochondrial mass that are in favor of CP resistance. The inhibition of PGC-1α or suppressing oxidative phosphorylation enhance CP sensitivity of NSCLC cells [116]. This experiment highlights the fact that we should consider metabolic reprogramming resulted from ROS and take strategies for overcoming this condition. The stimulation of factors involved in reducing ROS levels can promote CP resistance of NSCLC cells. Nrf2 participates in regulating redox balance and its activation is correlated with a decrease in ROS levels, and protecting cells against cell death [117,118]. Furthermore, Nrf2 activation can diminish ROS levels and prevent ferroptosis in cancer cells [119]. However, Nrf2 activation can diminish ROS levels in favor of inhibition of cell death in cancer cells and providing chemoresistance [98,120]. Such association has been examined in triggering CP resistance. It has been reported that polarity protein Scribble enhances CP sensitivity of NSCLC cells. However, in vitro and in vivo experiments have shown down-regulation of this factor in CP resistant-NSCLC cells. Upon Scribble down-regulation, proteasomal degradation of NADPH oxidase 2 (Nox2) occurs that subsequently, ROS levels decrease. On the other hand, Nrf2 signaling activation results from Scribble down-regulation that can also participate in decreasing ROS levels. These impacts together lead to the development of CP resistance in NSCLC cells and a reduction in CP-mediated apoptosis [121]. This experiment has potential application in clinical studies, since CP poses increasing challenges in the treatment of cancer patients, and if such signaling networks are affected in clinical course, we can prevent chemotherapy failure.

ROS inhibition can activate molecular pathways involved in cancer progression and phosphoinositide 3-kinase (PI3K)/protein kinase-B (Akt) is one of them. It has been reported that activation of PI3K/Akt axis not only promotes proliferation and metastasis of cancer cells [122,123,124,125], but also triggers chemoresistance [126,127,128,129]. Therefore, it is important to reveal the role of this molecular pathway in CP resistance of cancer cells and providing prospects for its targeting. In CP-resistant NSCLC cells, glutathione peroxidase 1 (GPX1) remarkably diminishes ROS levels to stimulate Akt signaling, as a tumor-promoting factor for CP resistance. The investigation of molecular pathways demonstrates that master transcription factor nuclear factor-kappaB (NF-κB) functions as upstream mediator of GPX1 in CP resistance, so that NF-kB inhibition leads to CP sensitivity of NSCLC cells [89]. GPX2 is also involved in CP resistance via reducing ROS levels, paving the way for failure of CP in lung cancer chemotherapy [130].

To be more specific about mechanisms involved in CP resistance, the significant role of drug transporters in this process should be considered and how they interact with ROS overgeneration. The enhanced activity of ATP-binding cassette (ABC) transporters such as multidrug resistance protein 1 (ABCB1) is suggested to induce CP resistance [111,131]. Importantly, revealing molecular pathways, regulating ABCB1 expression and activity, is of importance for providing a platform for next targeting in cancer treatment and enhancing CP sensitivity. It has been reported that EF hand domain-containing protein 2 (EFHD2) as a calcium-binding protein enhances production of NOX4 to promote ROS generation. Subsequently, ROS generation function as upstream mediator of ABCB1 to enhance its expression, resulting in CP resistance [132].

In the tumor microenvironment of cancer cells, some changes can occur to ensure progression and proliferation. The pyruvate kinase isoenzyme type M2 (PKM2) is a regulator of Warburg impact in cancer cells and can enhance glycolysis in cancer cells via catalyzing synthesis of pyruvate from phosphoenolpyruvate (PEP). Increasing evidence demonstrate the therapeutic potential of targeting PKM2 in cancer and enhancing CP sensitivity [133,134,135,136]. Exosomal transfer of PKM2 in hypoxic condition results in the generation of reductive metabolites that counter CP-mediated ROS production, preventing apoptosis and DNA damage and providing condition for CP resistance [137].

Thioredoxin (TRX1) is a disulfide-reducing dithiol enzyme and as an antioxidant enzyme plays a vital role in reduction of enzymes [138]. Recently, attention has been directed towards the role of TRX1 in cancer, particularly drug resistance. It has been reported that TRX inhibition inhibits drug resistance and viability of cancer cells via suppressing Akt phosphorylation and promoting caspase-3 expression [139]. Anti-tumor compounds, such as isodeoxyelephantopin are capable of down-regulating TRX1 and stimulating ROS-induced JNK signaling, leading to enhanced CP sensitivity [140]. Down-regulating TRX1 is suggested to promote dependency of cancer cells on oxidative metabolism. Furthermore, TRX1 down-regulation enhances ROS generation in cancer cells to increase their CP sensitivity [141].

One of the important aspects is the regulation of CP sensitivity by miRNAs [142]. Furthermore, miRNAs can modulate ROS levels in cells [143,144]. Therefore, understanding the role of miRNAs in regulating ROS levels in CP chemotherapy is significant. MiRNA-140 is a tumor-suppressing factor that enhances CP sensitivity of cancer cells via down-regulating Wnt signaling [97]. In increasing CP sensitivity, miRNA-140 down-regulates SIRT1 expression to promote ROS levels. Then, ROS induces JNK phosphorylation to increase CP-mediated apoptosis [113]. As more experiments are performed, different molecular pathways are revealed that mediate CP resistance of thoracic cancers. The tumor necrosis factor receptor-associated protein 1 (TRAP1) is a new therapeutic target in cancer. This mitochondrial heat shock protein can be found in other locations of cells such as nucleus, cytoplasm and endoplasmic reticulum [145,146]. It seems that upregulation of TRAP1 triggers drug resistance of cancer cells and prevents apoptosis [147]. The CP resistant-lung cancer cells demonstrate high expression level of TRAP1 and apoptosis inhibition. Silencing TRAP1 is associated with increase in capacity of CP in cancer elimination by enhancing ROS levels and mediating mitochondrial dysfunction [148]. 

In the introduction section, it was mentioned that ROS can induce apoptosis via triggering mitochondrial dysfunction. Furthermore, it was described that enhanced ROS overgeneration can enhance tumorigenesis. Such an association between ROS and mitochondrial dysfunction in enhancing gastric cancer progression has been evaluated. The eukaryotic initiation factor 2α (eIF2α)-ATF4 axis is a regulator of stress response and can provide conditions in favor of cell survival upon stressful conditions and preventing apoptosis [149,150]. There are different contributors of elF2a including dsRNA-activated protein kinase R (PKR), heme-regulated inhibitor eIF2α kinase (HRI), protein kinase R-like endoplasmic reticulum kinase (PERK), and general control nonderepressible-2 (GCN2) that are stimulated in various stress conditions [149]. When mitochondrial dysfunction occurs, GCN2 or PERK can enhance elF2α expression [151,152]. Exposing gastric cancer cells to CP increases expression level of SLC7A11 (×CT). It seems that mitochondrial dysfunction is responsible for enhanced ×CT and GSH expressions. Studies of the molecular pathways demonstrate that GCN2 can stimulate eIF2α/ATF4 axis to induce mitochondrial dysfunction, leading to enhanced ×CT and ROS levels, as well as triggering CP resistance [153]. Another experiment also reveals role of ×CT in CP resistance. However, in this study, upstream mediator of salubrinal plays an important. Salubrinal enhances expression level of ×CT to increase GSH expression, and silencing ×CT is associated with inability of salubrinal in triggering CP resistance, showing that ×CT is vital for this process. Furthermore, as ×CT enhances GSH expression, they may involve in reducing ROS levels and triggering CP resistance [154].

Noteworthy, molecular pathways that protect cancer cells against oxidative stress damage, can lead to CP resistance. Peroxiredoxin 2 (PRDX2) is a supporter of cells against oxidative damage via reducing ROS and H_2_O_2_ levels [155]. In gastric cancer cells, PRDX2 in cooperation with NF-kB-p65 subunit diminish ROS levels to suppress DNA damage and cell death, leading to CP resistance [156]. It seems that ROS participate in mechanisms that suppress CP-mediated apoptosis and mediate chemoresistance [157].

Recent years, much emphasis has been directed towards role of tumor microenvironment in cancer progression. Low levels of angiogenesis and high proliferation of cancer cells induce hypoxic conditions in the tumor microenvironment that are accompanied by an increase in expression level of hypoxia inducible factor-1α (HIF-1α) providing the conditions for cancer growth [158,159,160]. On the other hand, in response to different changes in the tumor microenvironment, alterations in structures and dynamics of mitochondria occur. The dynamin-related protein 1 (Drp1) is involved in mitochondrion dynamics and its phosphorylation level determines its activation or inhibition. For instance, Drp1 phosphorylation at serine 616 in results in its activation and mitochondrial fission, while phosphorylation at serine 637 prevents Drp1 activation and subsequent mitochondrial fission [161,162,163]. A recent study has clearly shed some light on the associations between mitochondria, hypoxia and CP resistance. In hypoxic conditions, an increase occurs in levels of ROS in ovarian cancer cells that subsequently, down-regulate the expression level of Drp1 (serine 637), resulting in mitochondrial fission and CP resistance. Furthermore, Mitofusins 1 and 2 (Mfn1 and 2) involving in mitochondrion dynamics are suppressed by hypoxia-mediated ROS to induce mitochondrial fission and CP resistance [164].

It is worth mentioning that ROS can associate metabolism and metastasis of cancer cells. Then, this relationship can be extended to even affect result of immunotherapy. Therefore, it is of great importance to understand ROS interaction with mechanisms involved in cancer metastasis and its association immune factors. Such relationships have been investigated in CP chemotherapy. It has been reported that high levels of ROS change the metabolic profile of lung cancer cells. This metabolism alteration leads to the reliance of lung cancer cells to mitochondrial oxidative metabolism than glucose. More investigations demonstrate that this metabolic alteration significantly enhances migration and invasion of lung cancer cells via EMT induction. Besides, EMT participates in triggering programmed death ligand-1 (PD-L1) upregulation that provides immune evasion of cancer cells [165]. This study clearly demonstrates that ROS, proliferation, metastasis and the response of cancer cells to chemotherapy and immunotherapy are in close relationship with each other, and ROS play the central and key role.

One of the pathways CP follow in cancer suppression is inducing DNA damage and preventing cancer progression. However, activation of signaling networks involved in DNA damage repair can provide CP resistance of cancer cells. Such phenomenon in ovarian cancer cells that can be targeted in next studies for triggering CP sensitivity. Dual oxidase 1 (DUOX1) is a carcinogenesis factor via increasing hydrogen peroxide levels [166]. Besides, DUOX1 can enhance ROS level to inhibit cell differentiation [167]. On the other hand, ataxia telangiectasia and Rad3-related protein (ATR) is a serine/threonine protein kinase modulating DNA damage [168]. It has been reported that ATR can induce Checkpoint kinase 1 (Chk1) to trigger DNA damage repair [169,170]. In ovarian cancer cells, DUOXA1 significantly elevates the production of ROS in stimulating ATR/Chk1 axis, leading to CP resistance. The in vitro and in vivo experiments have confirmed role of DUOXA1-mediated ROS overgeneration in CP resistance, and for overcoming poor prognosis in patients, targeting this pathway is of importance [171].

**Figure 2:**
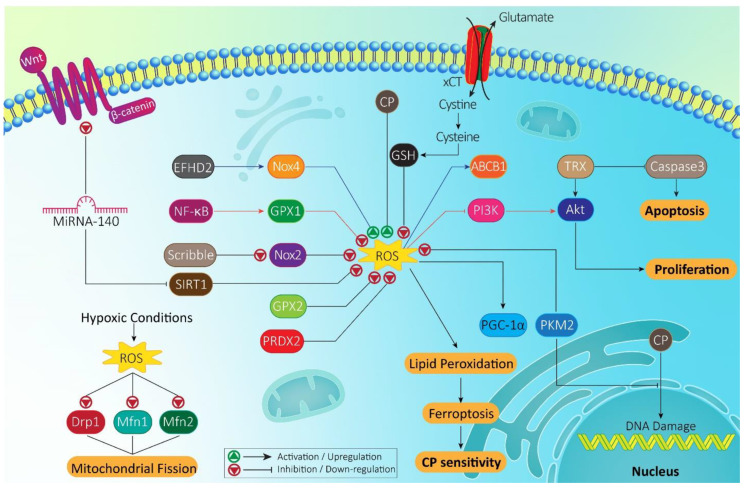
Molecular pathways regulating ROS in CP resistance. Mainly, ROS inhibition results in CP resistance, and upstream mediators including Nox2, GPX2, and SIRT1 can reduce ROS levels in mediating CP resistance. Furthermore, hypoxia affects ROS levels and mitochondrial function in CP resistance.

In previous sections, we discussed how Nrf2 signaling can participate in CP resistance. Another experiment also demonstrates role of Nrf2 signaling in CP resistance with an emphasis on upstream mediator of signaling. Increasing evidence shows tumor-promoting role of sirtuin-5 (SIRT5) in different cancers [172,173,174]. There is a dual relationship between SIRT5 and Nrf2 signaling in CP chemotherapy, so that SIRT5 can regulate Nrf2 signaling in reducing nephrotoxicity of CP [173]. In ovarian cancer cells, overexpression of SIRT5 is associated with CP resistance and prevents CP-mediated proliferation inhibition and DNA damage via reducing ROS levels. In this way, SIRT5 stimulates Nrf2 signaling and its downstream target heme oxygenase-1 (HO-1) to reduce ROS levels [175]. In fact, SIRT5/Nrf2 axis results in a reduction in ROS levels, and silencing SIRT5 or Nrf2 provides the way for CP sensitivity via ROS overgeneration. Another experiment also confirms how Nrf2 regulation by an upstream mediator can lead to CP resistance. In ovarian cancer cells with high expression level of p62, cancer cells are resistance to anti-tumor activity of CP. The investigation of molecular pathways demonstrates that p62 induces Nrf2 signaling via Keap1 down-regulation, resulting in reinforcement of antioxidant defense system and protection of cancer cells against inhibitory impact of CP [176]. It has been reported that ROS can function as upstream mediator of tumor-promoting factors in CP resistance. Previously, we described the role of PGC-1α in CP resistance. In ovarian cancer cells, mitochondrial dysfunction enhances ROS levels to stimulate PGC-1α expression, leading to CP resistance [177]. As more experiments are performed, more signaling networks involved in CP resistance of ovarian cancer cells are revealed [178]. Figure 2 and Table 2 provide a summary of ROS and related molecular pathways in CP resistance.

## 5. Therapeutic Targeting

In respect of the fact that molecular pathways involved in CP resistance and their regulatory impact on ROS levels and signaling have been identified, experiments have focused on using anti-tumor compounds, which are mostly phytochemicals. In the section, we provide a mechanistic discussion around using these compounds and their signaling targets. Plant derived-natural compounds have opened a new gate in cancer therapy due to their multitargeting capacity [193,194,195,196]. Melatonin is a hormone of pineal gland that is synthesized in other organs with higher concentrations [197]. Recent studies have shown different biological and therapeutic activities of melatonin that anti-tumor activity is among them. Noteworthy, melatonin can be considered as a potent chemosensitizer agent [198]. In this way, melatonin can also enhance anti-tumor activity of CP. For instance, it has been reported that melatonin can activate caspase-3/7 cleavage and induce cell cycle arrest in potentiating cytotoxicity of CP against lung cancer cells [199]. Importantly, ROS plays a key role in mediating anti-tumor activity of melatonin and its capacity in promoting CP sensitivity. By enhancing ROS levels, melatonin activates intrinsic pathway of apoptosis, resulting in enhanced CP sensitivity of cervical cancer cells [200]. In addition to apoptosis, melatonin can affect other pathway of programmed cell death, known as autophagy. Generally, autophagy is a “self-digestion” mechanism and its induction is of importance in cancer therapy [201,202]. Increasing evidence demonstrate the close relationship between autophagy and ROS, so that ROS overgeneration can stimulate autophagy [203,204]. By enhancing ROS levels, melatonin simultaneously induces autophagy and apoptosis [205]. A similar strategy is followed by withaferin-A in enhancing CP sensitivity of oral cancer cells via enhancing ROS levels and triggering both apoptosis and autophagy [206]. However, one hint should be considered that autophagy may stimulate chemoresistance [207], and when investigating dual relationship between autophagy and ROS, this aspect of autophagy should be highlighted and considered.

Emodin is a plant derived-natural compound with high anti-tumor activity [208,209]. This potent anti-tumor agent can suppress cancer metastasis via inhibiting epithelial-to-mesenchymal transition (EMT) [204]. The anti-tumor activity of emodin is dose-dependent and can affect different molecular pathways, such as miRNA-34a and vascular endothelial growth factor receptor (VEGFR) [210]. In enhancing CP sensitivity of endometrial cancer cells, emodin targets ROS levels. In this way, emodin diminishes ROS levels to induce apoptosis and suppress tumor growth (both in vitro and in vivo) [211]. Another experiment also confirms the role of emodin in increasing ROS levels, and potentiating the anti-tumor activity of CP against bladder cancer cells [212]. In fact, several signaling networks are affected by anti-tumor compounds in triggering CP sensitivity that enhancing ROS levels is one of them [213].

Previously, it was shown that Nrf2 signaling activation is in favor of CP resistance via reducing ROS levels. Noteworthy, anti-tumor compounds targeting Nrf2 signaling and enhancing CP sensitivity have been discovered. Exposing head and neck cancer cells to wogonin, as a flavonoid compound, significantly reduces expression level of Nrf2, leading to CP sensitivity through increasing ROS accumulation [214]. Another experiment also reveals the down-regulation of Nrf2 upon CP and a novel polyphenol, known as (E)-3-(3,5-dimethoxyphenyl)-1-(2-methoxyphenyl)prop-2-en-1-one (DPP-23), to enhance ROS accumulation, resulting in cell death and increased CP sensitivity [215]. However, we still have a long way in regulating Nrf2 signaling, since this study has just examined the expression level of Nrf2. What about anti-tumor compounds targeting Keap1 or nuclear translocation of Nrf2? Future experiments will appropriately respond to this question.

Allicin is another naturally occurring compound with the capacity to suppress cancer proliferation, increase radio-sensitivity, and down-regulate NF-κB signaling [216]. Allicin is extensively applied with other chemotherapeutic agents. For instance, allicin can promote chemosensitivity of cancer cells via apoptosis induction, enhancing miRNA-486-3p level and reducing cancer cell viability [217,218]. A newly conducted experiment has obviously demonstrated the role of allicin in CP sensitivity of lung cancer cells. In this way, allicin increases ROS levels to induce both autophagy and apoptosis, and trigger cell cycle arrest (S/G2-M phase) [219]. By increasing ROS levels, a decrease occurs in intracellular level of GSH that is in favor of apoptosis induction via caspase-3 and -7 stimulation [220]. Previously, it was discussed that Akt phosphorylation and activation can promote cancer progression and induce chemoresistance [221,222]. Interestingly, ROS can function as an upstream mediator of Akt signaling [223,224]. Piperlongumine as an anti-tumor agent, promotes ROS levels and accumulation in lung cancer cells to suppress Akt signaling, leading to CP sensitivity [225]. Another aspect is related to impact of anti-tumor compounds on CP-mediated DNA damage, so that by increasing ROS levels, anti-tumor compounds enhance p53 phosphorylation to induce DNA damage and cell death [226]. The importance is efficacy of this combination in enhancing anti-tumor activity of CP in vivo, so that the combination of CP and shikonin effectively suppresses tumor growth in colon cancer (HCT116 xenograft tumor) [227]. Therefore, the next step can be translating these findings to clinical application for enhancing the overall survival of cancer patients and preventing chemotherapy failure.

Clarithromycin (CAM) is a well-known antibiotic that was first applied in 2005. CAM can affect both apoptosis and autophagy by enhancing cytotoxicity of 5-fluorouracil as a chemotherapeutic agent against colorectal cancer cells [228]. A similar phenomenon occurs during CP chemotherapy. In this way, CAM significantly enhances ROS levels to impair ovarian cancer growth in vitro and in vivo, leading to CP sensitivity [229]. However, the story is not always so simple. The dual role of ROS as a pro-survival and pro-death mechanism was extensively discussed in the introduction section. AXL is a receptor tyrosine kinase with a role in cancer that has been suggested to be tumor-promoting. In increasing metastasis of breast cancer cells and providing their immune evasion, AXL and Mertk cooperate together [230]. It has been reported that the overexpression of AXL can induce mitogen-activated protein kinase (MAPK) and triggering therapy resistance [231]. In ovarian cancer cells, decreasing AXL expression is correlated with CP sensitivity by suppressing glycolysis [232]. A combination of CP and pemetrexed can sufficiently stimulate cell death in mesothelioma cells via enhancing ROS levels. However, ROS signaling activates AXL, which diminishes cytotoxicity against cancer cells. In providing effective cancer chemotherapy, it is better co-administer a AXL blocker such as BGB324 with CP and pemetrexed [233]. This study reminds us that although anti-tumor compounds enhance ROS production in providing CP sensitivity, it should be noted that ROS can activate downstream targets with tumor-promoting roles such as AXL.

**Figure 3:**
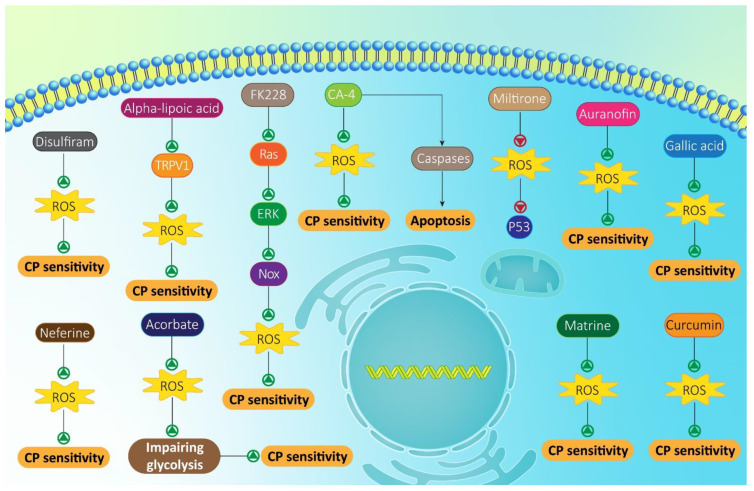
Anti-tumor compounds targeting ROS and mediating CP sensitivity. Most of them are phytochemical and mainly enhance ROS levels in apoptosis induction and promoting potential of CP in cancer suppression.

It is worth mentioning that CP can promote ROS levels in mediating cell death in cancer cells. However, when an anti-tumor agent, such as vitamin D is co-administered with CP, its potential in enhancing ROS levels enhances [234]. Furthermore, a combination of CP with other anti-tumor compounds provide conditions for suppressing molecular pathways that can enhance cancer progression. For instance, plumbagin and CP induce JNK signaling, while they inhibit Akt/mTOR signaling to enhance ROS levels, leading to apoptosis, autophagy and decreased viability of tongue squamous cell carcinoma cells [235]. NF-κB signaling pathway is a molecular pathway where overexpression paves the way for chemoresistance of cancer cells [206,236]. Triptolide promotes intracellular accumulation of ROS to inhibit NF-κB signaling and down-regulate Bcl-2 and X-linked inhibitor of apoptosis protein (XIAP) as anti-apoptotic factors, increasing CP sensitivity of ovarian cancer cells [237]. Reducing glycolysis (Warburg effect) and impairing mitochondrial function are induced by ascorbate in increasing CP sensitivity of osteosarcoma cells (Figure 3) [238]. Overall, the following points can be concluded about using anti-tumor compounds, which are mostly phytochemicals and have roles in enhancing CP sensitivity of cancer cells:

- Anti-tumor compounds significantly promote intracellular accumulation of ROS to mediate intrinsic pathway of apoptosis via mitochondrial dysfunction [239,240,241,242,243,244,245,246,247,248,249,250],
- Molecular pathways responsible for cancer progression and mediating CP resistance are suppressed by anti-tumor compounds upon increasing ROS levels [251,252,253,254],
- Most of the anti-tumor compounds applied with CP in cancer chemotherapy are plant derived-natural products, and one of their drawbacks is their poor bioavailability that can be overcome using nanoparticles. This aspect is discussed in next section (Table 3 and Table 4).

## 6. Gene Therapy

In relation to the fact that molecular pathways, responsible for CP resistance, have been identified, genetic tools can be employed in providing CP sensitivity. This strategy can be specified by targeting molecular pathways that regulate ROS in CP chemotherapy. Although a few experiments have evaluated role of gene therapy in affecting ROS and CP sensitivity, this section provides a mechanistic discussion with future prospects to show how genetic tools can be utilized for affecting ROS and CP sensitivity.

Previously, it was mentioned that HIF-1α is activated in hypoxic conditions and can promote cancer progression [294,295,296,297,298,299]. As there is a close relationship between HIF-1α and cancer metabolism, targeting this molecular pathway is of importance in CP sensitivity. Among genetic tools, small interfering RNA (siRNA) has shown high potential in promoting CP sensitivity via down-regulating tumor-promoting factors [296,300,301]. In this case, HIF-1α down-regulation by siRNA leads to a change in cancer metabolism from aerobic glycolysis to mitochondrial oxidative phosphorylation. Then, ROS overgeneration occurs, resulting in apoptosis and increased CP sensitivity. This experiment obviously demonstrates impact of siRNA on ROS-related molecular pathways and their role in CP chemotherapy. Furthermore, in order to promote the potential of siRNA in gene silencing, its delivery by attenuated Salmonella has been performed [302]. In addition to HIF-1α, Nrf2 signaling role in CP resistance has been discussed before [303]. It seems that down-regulating Nrf2 expression by siRNA paves the way for CP sensitivity via inhibiting HO-1, subsequent increase in ROS generation and promoting CP-mediated cell death [304]. Future experiments can focus on developing nanoparticles for siRNA delivery, affecting molecular pathways regulating ROS and promoting CP sensitivity. More experiments are needed to target factors regulating ROS levels in CP chemotherapy, paving the way for cancer elimination. Furthermore, other kinds of genetic tools, such as CRISPR/Cas9 system and short-hairpin RNA (shRNA) can be utilized in this case. 

## 7. Nanotherapeutics

In the previous section, a mechanistic discussion of the role of molecular pathways regulating ROS levels in CP resistance/sensitivity was provided. Then, it was shown that anti-tumor compounds can affect ROS levels in mediating CP sensitivity. However, these therapies suffer from poor bioavailability and provide a platform for their targeted delivery is important in increasing their efficacy in triggering CP sensitivity. Furthermore, upstream mediators of ROS can be targeted by genetic tools, such as siRNA. However, siRNA should first circulate in blood and then move to the tumor site. It may be degraded by enzymes, while circulating in blood, and also, its efficacy increases by targeted delivery thereby promoting its intracellular accumulation [305,306]. In this section, we demonstrate how nanocarriers can be helpful in regulating ROS levels and providing CP sensitivity.

Nanoscale delivery systems can significantly promote intracellular accumulation of drugs in cells via mediating endocytosis [307,308]. Another benefit of using nanocarriers is providing simultaneous chemotherapy and phototherapy in cancer eradication [309,310]. Such a strategy has been applied for CP delivery and preventing drug resistance. In this case, mesoporous silica nanoparticles (MSNs) have been developed for CP delivery. In order to provide phototherapy capacity of MSNs, their surface modification by chlorin e6 (Ce6) was performed. The nanocarriers demonstrated good properties such as particle size of 100 nm and zeta potential of 18.2 mV. These nanoparticles penetrate into cancer cells through endocytosis to promote intracellular accumulation of CP. Exposure to 660 nm light irradiation induces phototherapy effect and significantly promote ROS production in lung cancer cells, leading to enhanced efficacy of CP in cancer elimination [311]. Another experiment also demonstrates the role of photodynamic therapy in increasing ROS levels, and sensitizing cancer cells to apoptosis that are of importance in promoting their CP sensitivity [312]. Overall, irradiation and photo-excitation are vital for promoting ROS levels and activating pro-apoptotic factors, such as p38 MAPK to increase CP sensitivity of cancer cells [313]. It is worth mentioning that nanoparticles can also mediate co-delivery of CP with other anti-tumor compounds. Metformin is a potent anti-tumor compound that suppresses mammalian target of rapamycin (mTOR) via AMP-activated protein kinase (AMPK) upregulation, leading to CP sensitivity of cancer cells [314]. For enhancing the efficacy of metformin and CP in cancer chemotherapy, nanoplatforms have been developed [315]. It is worth mentioning that metformin- and CP-loaded nanoparticles can affect ROS. In this way, exposing colorectal cancer cells to CP- and metformin-loaded nanocubosomes is associated with an increase in ROS levels, that subsequently, enhance NADPH oxidase, while decreasing lactate dehydrogenase (LDH), leading to caspase-3 cleavage and chemosensitivity [316].

Curcumin is also a plant derived-natural compound with diverse therapeutic effects that anti-tumor activity is among them [317,318,319,320,321]. Curcumin is extensively applied with CP in suppressing progression of cancer cells and providing their chemosensitivity via targeting molecular pathways and mechanisms such as apoptosis, metastasis, KLF4 and SOX2 [322,323]. Loading CP and curcumin on liposomal nanocarriers increases their potential in enhancing ROS levels and suppressing hepatocellular carcinoma progression [324]. Another experiment also reveals role of curcumin-loaded nanoparticles in increasing ROS levels in oral cancer cells and sensitizing them to CP-mediated cell death [325]. In fact, the field of materials science can direct us towards using agents capable of promoting ROS levels and reversing CP resistance. Such a strategy has been utilized recently by Sun and colleagues. In this way, they synthesized nanogel by conjugating chitosan to diallyl disulfide, and then, its grafting with valproate. The interesting point is that valproate induces 18-fold increase in p53 expression, and simultaneously, diallyl disulfide triggers 8-fold increase in ROS levels, leading to CP sensitivity. Furthermore, in vivo experiment also confirmed role of this nanogel in reducing tumor growth inhibition and CP sensitivity [326]. A newly conducted experiment demonstrates that tocotrienols-, caffeic acid- and CP-loaded nanoemulsions can enhance ROS production up to 16.9%, and 30.2% in lung and liver cancers, respectively [327], that are importance in mediating cell death and preventing cell cycle progression.

Notably, carbon nanomaterials, such as graphene possess carcinogenesis impact [296]. Applying such carriers for CP delivery may exert reverse effect and promote drug resistance of cancer cells. It has been reported that CP-loaded multiwalled carbon nanotubes significantly diminish ROS levels and induce failure of CP in mediating apoptosis in breast cancer cells, leading to development of drug resistance [328]. Therefore, this aspect should be considered while synthesizing nanocarriers for CP delivery and suppressing cancer progression.

**Figure 4:**
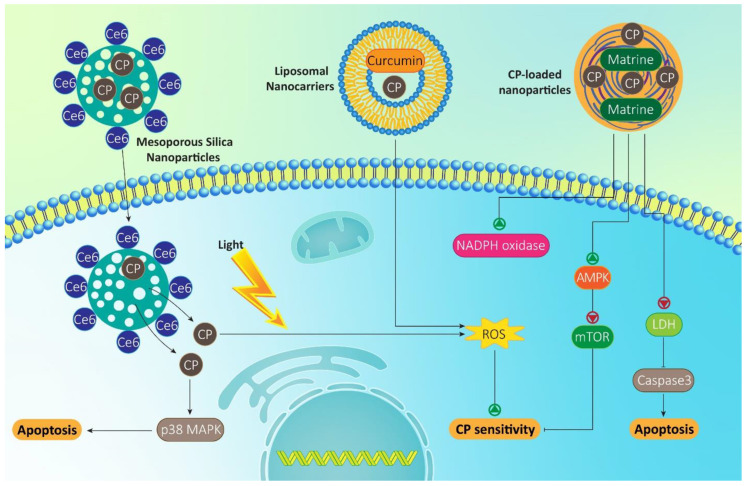
Nanoscale delivery systems in ROS regulating and CP sensitivity. Nanoparticles enhance penetration of CP through cell membrane and via increasing intracellular accumulation, promote its potential in ROS overgeneration and cancer cell death. Anti-tumor compounds such as curcumin and matrine can be co-delivered by CP in effective cancer suppression. Furthermore, phototherapy mediated by nanoparticles enhances CP sensitivity of cancer cells.

Overall, studies are in line with the fact that using nanoparticles is of importance in increasing ROS levels and sensitizing cancer cells to CP chemotherapy. Furthermore, nanocarriers can undergo surface modification to enhance their selectivity towards cancer cells. Finally, nanoparticles can provide phototherapy in promoting ROS generation, resulting in an increase in efficacy of CP in cancer chemotherapy (Figure 4) [298,329,330,331].

## 8. Conclusions and Remarks

In the present review, a comprehensive discussion of ROS role in CP resistance/sensitivity was provided. Due to frequent application of CP, cancer cells have obtained resistance to this chemotherapeutic agent, and if an effective cancer chemotherapy is performed, molecular pathways and mechanisms responsible for CP resistance should be identified so they can be targeted through novel therapeutics. The exact role of ROS in cancer cells has not been completely determined, and it may act as a pro-survival or pro-death mechanism. This context-dependent role of ROS has resulted in much attention in revealing its role in CP resistance/sensitivity. Upstream mediators of ROS can affect response of cancer cells to CP chemotherapy, and noteworthy, downstream targets also play a significant role, as shown in this review. The important hint is that experiments have used therapeutic agents in targeting ROS and providing CP sensitivity. In this case, both genetic and pharmacological interventions have been performed. Anti-tumor compounds that are mostly phytochemicals, enhance ROS levels to mediate mitochondrial dysfunction and cell death. It should be noted that ROS can activate both autophagy and apoptosis. In contrast to apoptosis, autophagy can promote the progression of cancer cells [332]. Therefore, if autophagy activation occurs following pharmacological intervention and enhancing ROS levels in CP chemotherapy, the exact role of autophagy should be determined, and if autophagy functions as a pro-survival mechanism, autophagy inhibitors, such as chloroquine can be utilized.

Another important aspect is using gene therapy to influence levels and CP chemotherapy. Similar to pharmacological intervention, genetic tools can also promote CP sensitivity via regulating ROS levels. However, the drawbacks of these strategies should also be considered. For instance, anti-tumor compounds suffer from poor bioavailability. Genetic tools, such as siRNA may undergo degradation while circulating in blood and it has an off-targeting feature. To overcome the aforementioned disadvantages, scientists have focused on developing nanoarchitectures. These nanocarriers provide targeted delivery, co-delivery with other anti-tumor agents and genetic tools, increased intracellular accumulation in cancer cells and promote ROS generation that are important in CP sensitivity. Although pre-clinical studies have investigated ROS and CP chemotherapy, future experiments can focus on developing novel therapies for targeting ROS in the treatment of cancer patients. Furthermore, if nanoparticle application is applied in this field, a biocompatibility profile should be considered.

## Figures and Tables

**Table 1:** Enhanced CP sensitivity of cancer cells via ROS regulation.

Cancer Type	In vitro/In vivo	Cell line/Animal Model	Signaling Network	Remarks	Refs
Osteosarcoma	In vitro	MG63/DDP and Saos-2/DDP cells	STAT3/Nrf2/GPX4	High expression of STAT3, Nrf2 and GPX4 in CP resistant-cancer cells STAT3 inhibition promotes CP sensitivity Agonist of ferroptosis enhances CP sensitivity ROS overgeneration partially is involved in triggering CP sensitivity	[108]
Sarcoma	In vitro	MG-63 cells	Id3/ROS	Enhancing Id3 expression increases CP sensitivity of cancer cells by apoptosis induction via ROS overgeneration	[109]
Human maxillary cancer	In vitro	IMC-3CR cells	SESN1/ROS	Reducing apoptosis induction Enhancing viability and survival of cancer cells SENS1 decreases ROS levels	[110]
Tongue squamous cell carcinoma	In vitro	CAL27 cells	-	ROS overgeneration enhances anti-tumor activity of CP Simultaneous induction of apoptosis and autophagy	[111]
Ovarian cancer	In vitro	OVCAR-3 cells	-	Higher levels of mitochondrial ROS in CP sensitive-cancer cells compared to CP resistant-cancer cells Boosting CP-mediated apoptosis via enhancing ROS levels	[112]
Non-small cell lung cancer	In vitro	A549 cells	MiRNA-140/SIRT1/ROS/JNK	MiRNA-140 functions as a tumor-suppressing factor SIRT1 down-regulation Activating ROS/JNK axis Increasing CP sensitivity	[113]
Breast cancer	In vitro	MCF-7 cells	ACO2/ROS	ACO2 promotes ROS accumulation in cancer cells Subsequent stabilization and stimulation of p53 in nucleus and mitochondria Apoptosis induction	[114]
Colorectal cancer	In vitro	HT29 and SW480 cells	MiRNA-519d/TRIM32	Down-regulating TRIM32 by miRNA-519d Promoting CP sensitivity via ROS generation, and mediating mitochondrial pathway of apoptosis	[94]
Colon cancer	In vitro	HCT-15 cells	-	Reduced levels of ROS Down-regulation of KLF4 CP resistance	[86]
Hepatocellular carcinoma	In vitro	HepG2 and Huh7 cells	MiRNA-124/SIRT1/ROS/JNK	SIRT1 inhibition Triggering JNK phosphorylation via ROS overgeneration Mediating CP sensitivity	[91]

**Table 2:** Experiments related to CP resistance and role of ROS generation.

Cancer Type	In Vitro/In Vivo	Cell Line/Animal Model	Signaling Network	Remarks	Refs
Urothelial carcinoma	In vitro	T24 and UMUC3 cells	MUC1-C/xCT/GSH	Reducing ROS levels MUC1 enhances expression level of xCT to promote GSH level Inducing CP resistance	[179]
Squamous cell carcinoma	In vitro	EC109 cells	MUTYH/ROS	Down-regulation of MUTYH occurs in CP resistant cancer cells MUTYH down-regulation is associated with decreased levels of ROS	[180]
Oral squamous cell carcinoma	In vitro	Tca8113 cells	SIRT1/ROS	Reducing ROS accumulation in cancer cells Inducing CP resistance	[181]
Bladder cancer	In vitro	HT1376 cells	AKR1C2/ROS	Reducing AKR1C2 expression promotes CP sensitivity of cancer cells, determining oncogene role of this factor AKR1C2 diminishes ROS levels in mediating CP resistance	[182]
Bladder urothelial carcinoma	In vitro	NTUB1 cells	CEBPD/ROS	Upregulation of CEBPD in Cp resistant-cancer cells Decreasing ROS levels Apoptosis inhibition	[183]
Osteosarcoma	In vitro	MG63, U2OS and 143B cells	TERT/ROS	Telomerase diminishes ROS levels in cells Reducing apoptosis Improving mitochondrial function Inducing CP resistance	[184]
Osteosarcoma	In vitro	U2OS, SAOS2, MG-63 and HOS cells	APE1/ROS	Overexpression of APE1 is observed in CP resistant-osteosarcoma cells APE1 upregulation diminishes apoptosis and DNA damage Preventing ROS generation by APE1 upon exposure to CP	[185]
Different cancers	In vitro	293T, Caov-3, BG-1, and KB-3-1 cells	IP4/NOX4/ROS	Inhibition of NOX4 by IP4 Reducing ROS levels Triggering CP resistance	[186]
Different cancers	In vitro	H1299 and P31 cells	SIRT3/ROS HIF-1α/ROS	Increased levels of ROS in CP resistant-cancer cells, showing oncogene role of ROS Simultaneous upregulation of HIF-1α with ROS overgeneration SIRT3 down-regulation with simultaneous ROS overgeneration	[187]
Ovarian cancer	In vitro	SKOV3 cells	P62/Keap1/Nrf2/ARE	Upregulation of p62 in CP resistant-ovarian cancer cells Induction of Nrf2/ARE axis via Keap1 down-regulation Reducing ROS levels Preventing apoptosis	[176]
Ovarian cancer	In vitro	SKOV3 and A2780 cells	RIP1/ROS	Acting as a tumor-promoting factor via reducing ROS accumulation Enhancing ROS accumulation promotes apoptosis and necroptosis in cancer cells	[188]
Human mesothelioma	In vitro	ZL55 cells	ROS/PKC-α/EGFR/ERK1/2	CP induces ROS overgeneration that in turn, stimulates PKC-α Activation of EGFR and subsequent phosphorylation of ERK1/2 are responsible for reduced CP cytotoxicity against cancer cells	[189]
Non-small cell lung cancer	In vitro	H460 cells	ROS/CAV1	ROS overgeneration upon sub-toxic exposure to CP results in CAV1 upregulation and anoikis resistance, reducing efficacy of chemotherapy	[190]
Glioma	In vitro	U251 cells	ROS/Akt/mTOR	Inducing Akt/mTOR signaling via ROS overgeneration Promoting autophagy Triggering CP resistance Reducing ROS levels inhibit Akt signaling, showing role of ROS in CP resistance	[191]
Gastric cancer	In vitro	SNU-16 cells	-	Enhancing ROS levels Inducing Akt signaling Providing CP resistance Upregulating p53 expression suppresses CP resistance of cancer cells	[192]

**Table 3:** Anti-tumor compounds applied in regulating ROS levels and enhancing CP sensitivity.

Anti-Tumor Compound	Cancer Type	In Vitro/In Vivo	Cell Line/Animal Model	Study Design	Signaling Network	Remarks	Refs
Disulfiram	Breast cancer	In vitro	MCF-7, SKB-R3, and MDA-MB-435S cells	1 µM 24 h	-	Enhancing ROS levels Potentiating cytotoxicity of CP against breast cancer cells	[255]
FK228	Breast cancer	In vitro	MCF10A cells	0–1 nM	ERK/NOX/ROS	Stimulating ERK/NOX axis via affecting Ras signaling Increasing intracellular accumulation of ROS in cells Mediating cell death and apoptosis Enhancing CP sensitivity of cancer cells	[256]
CA-4 (microtubule inhibitor)	Lung cancer	In vitro	A549 cells	0.21 µM	-	Enhancing ROS generation Subsequent loss in mitochondrial membrane potential Activating apoptosis through inducing caspase cascade Enhancing CP sensitivity	[257]
LW6 (HIF-1α inhibitor)	Non-small cell lung cancer	In vitro	A549 cells	0–96 h	-	Suppressing hypoxia-mediated resistance to CP chemotherapy Increasing ROS levels Decreasing MRP1 and MDR1 levels Triggering CP sensitivity	[258]
4-phenylbutyrate	Ovarian cancer	In vitro	A2780 cells	0–50 µM	-	Increasing ROS generation Inhibiting activity of histone deacetylase Inducing apoptosis and DNA damage	[259]
ABT737	Ovarian cancer	In vitro	SKOV3 cells	0–40 µM	-	Down-regulating Bcl-2 expression Impairing glucose metabolism Potentiating anti-tumor activity of CP	[260]
Brown algae phlorotannins	Ovarian cancer	In vitro In vivo	A2780 and SKOV3 cells Mouse model	75 and 150 mg/kg	ROS/Akt/NF-κB	Increasing ROS levels and subsequent inhibition of Akt/NF-κB axis Inducing cell death and tumor growth inhibition in vitro and in vivo	[261]
Bithionol	Ovarian cancer	In vitro	A2780 /A2780-CDDP and IGROV-1/, IGROV-1CDDP cells	12.5 µM	-	Triggering ROS-mediated apoptosis Down-regulation of XIAP, Bcl-2 and Bcl-Xl as pro-survival factors Upregulating PARP, and caspase-3/7 as pro-apoptotic factors Triggering cell cycle arrest via p21 and p27 upregulation	[262]
Emodin	Ovarian cancer	In vitro	COC1 cell line	12.5, 25 and 50 µM	ROS/MRP1	Down-regulating MRP1 expression via ROS overgeneration Promoting CP sensitivity	[263]
Metformin	Colorectal cancer	In vitro	SW480 and SW620 cells	0–20 mM	ROS/PI3K/Akt	Inducing ROS overgeneration Subsequent inhibition of PI3K/Akt signaling Increasing CP sensitivity	[264]
Benzyl Isothiocyanate	Leukemia	In vitro	HL-60 cells	0–5 µM	-	Reducing GSH levels Inducing ROS overgeneration Promoting cell death Providing CP sensitivity Triggering ERK signaling pathway	[265]
Chloroquine	Cholangiocarcinoma	In vitro	QBC939 cells	50 µM	-	Reducing G6PDH activity Promoting ROS accumulation Autophagy inhibition Sensitizing to cell death and enhancing CP sensitivity	[266]
Chloroquine	Urothelial cancer	In vitro	NTUB1 and N/P (cisplatin-resistant sub-line) urothelial cancer cells	10 µM	ROS/LC-3II	Enhancing ROS generation ROS scavenger reduces LC-3II accumulation, showing role of ROS in upregulating LC-3II levels Inducing cell death independent of caspase and based on autophagy Increasing CP sensitivity	[267]

**Table 4:** Plant derived-natural compounds regulating ROS levels in CP chemotherapy.

Anti-Tumor Compound	Cancer Type	In Vitro/In Vivo	Cell Line/Animal Model	Study Design	Signaling Network	Remarks	Refs
Alpha-lipoic acid	Breast cancer	In vitro	MCF-7 cells	0.05 mM	TRPV1/ROS	Inducing TRPV1 and subsequent increase in ROS levels Decreasing viability and proliferation of cancer cells Enhancing CP sensitivity	[268]
Neferine	Lung cancer	In vitro	A549 cells	10 µM	-	Enhancing ROS levels Inducing mitochondrial dysfunction Apoptosis induction	[269]
Miltirone	Lung cancer	In vitro	A549 cells	0–40 µM	-	Reducing ROS levels to promote p53 expression, demonstrating oncogene role of ROS	[270]
Bu-Zhong-Yi-Qi Decoction	Lung cancer	In vitro	A549 cells	0–5000 µg/ml	ROS/Apoptosis ROS/Autophagy	Enhancing ROS generation and inducing cell death, both autophagy and apoptosis ROS scavenger reduces cell death, showing role of ROS in CP-mediated cell death in cancer cells	[271]
Auranofin	Lung cancer	In vitro In vivo	H69 and H196 cells Xenografts	500 and 1000 nM 10 mg/kg	-	Inducing ROS overgeneration Triggering mitochondrial dysfunction Enhancing DNA damage Suppressing tumor growth in vivo Increasing CP sensitivity	[272]
Gallic Acid	Small cell lung cancer	In vitro	H446 cell line	3 µg/mL 24 h	-	Suppressing cancer growth Apoptosis induction Enhancing ROS levels	[273]
Osthole derivative	Lung cancer	In vitro	A549 cells	0–10 µM	-	Triggering oxidative stress via ROS overgeneration Enhancing CP sensitivity	[274]
Yu Ping Feng San	Lung cancer	In vitro In vivo	A549 cells Tumor-bearing mice	0–20 µM 4 g/kg	-	Decreasing tumor volume Reducing cancer cell viability Increasing ROS levels Promoting CP sensitivity	[275]
Curcumin	Bladder cancer	In vitro	253J-Bv cells	10 µM	ROS/ERK1/2	Enhancing ROS levels to induce ERK1/2 Apoptosis induction Providing CP sensitivity	[276]
Matrine	Urothelial bladder cancer	In vitro	EJ, T24, BIU, 5637 cells	1–16 mM	-	Increasing ROS generation and sensitizing cancer cells to apoptosis Promoting CP sensitivity	[277]
β-elemene	Bladder cancer	In vitro	T24 and 5637 cells	0–75 µg/ml	ROS/AMPK	Preventing cancer cell proliferation Triggering cell cycle arrest (G0/G1 phase) Increasing intracellular accumulation of ROS Stimulating AMPK signaling Apoptosis induction	[278]
Osthole derivative	Lung cancer	In vitro	A549 cells	0–10 µM	-	Triggering oxidative stress via ROS overgeneration Enhancing CP sensitivity	[274]
Yu Ping Feng San	Lung cancer	In vitro In vivo	A549 cells Tumor-bearing mice	0–20 µM 4 g/kg	-	Decreasing tumor volume Reducing cancer cell viability Increasing ROS levels Promoting CP sensitivity	[275]
Curcumin	Bladder cancer	In vitro	253J-Bv cells	10 µM	ROS/ERK1/2	Enhancing ROS levels to induce ERK1/2 Apoptosis induction Providing CP sensitivity	[276]
Matrine	Urothelial bladder cancer	In vitro	EJ, T24, BIU, 5637 cells	1–16 mM	-	Increasing ROS generation and sensitizing cancer cells to apoptosis Promoting CP sensitivity	[277]
β-elemene	Bladder cancer	In vitro	T24 and 5637 cells	0–75 µg/mL	ROS/AMPK	Preventing cancer cell proliferation Triggering cell cycle arrest (G0/G1 phase) Increasing intracellular accumulation of ROS Stimulating AMPK signaling Apoptosis induction	[278]
Withaferin A	Ovarian cancer	In vitro	A2780 and A2780/CP70 cells	0–7 µM	-	Inducing DNA damage through promoting ROS levels and sensitizing cancer cells to CP chemotherapy	[279]
Cucurbitacin B	Ovarian cancer	In vitro	A2780 cells	0–8 µM	-	Significant decrease in viability and proliferation of cancer cells Increasing their sensitivity to CP Promoting ROS production	[280]
Curcumin	Laryngeal squamous cell cancer	In vitro	Hep2 cells	1 µM	-	CP administration enhances ROS levels to induce apoptosis in cancer cells Combination chemotherapy with curcumin increases TRPM2 level to potentiate cytotoxicity against cancer cells and enhance efficacy of CP in increasing ROS levels	[281]
Asteriscus graveolens	Lymphoma	In vitro	BS-24-1 cells	0–8 µg/ml	-	Enhancing ROS levels Sensitizing cancer cells to CP-mediated apoptosis	[282]
Zinc protoporphyrin IX	Liver cancer	In vitro	HepG2 cells	10 µmol/L	HO-1/ROS	Down-regulating HO-1 expression Increasing ROS levels Activating caspase-3 Sensitizing to CP-mediated cell death	[283]
Tigecyclin	Hepatocellular carcinoma	In vitro	HepG2 and HuH6 cells	1, 5 and 10 µM	-	Inducing oxidative stress through ROS overgeneration Decreasing mitochondrial respiration Increasing CP sensitivity	[284]
α-Hederin	Gastric cancer	In vitro In vivo	SGC-7901, HGC-27, and MGC-803 cells Xenograft mouse model	4 mg/kg	-	Enhancing tumor growth inhibition capacity of CP in vivo Promoting expression level of apoptosis proteins Increasing ROS levels	[285]
α-Hederin	Gastric cancer	In vitro In vivo	HGC27 cells Nude mice	0-25 µM 2, 4 and 6 mg/kg	-	Apoptosis stimulation Triggering GSH depletion Increasing intracellular accumulation of ROS	[286]
Docosahexaenoic acid	Gastric cancer	In vitro	SNU-601 cells and SNU-601/cis2 cells	0-200 µM	GPR120	GPR120 mediates capacity of DHA in increasing ROS levels and inducing apoptosis in cancer cells	[287]
Oxymatrine	Gastric cancer	In vitro	BGC-823 and SGC7901 cells	1 mg/mL	Akt/ERK	Inducing apoptosis in cancer cells in a ROS-dependent manner Suppressing Akt/ERK axis Upregulating p21 and p27 levels	[288]
Resveratrol	Mesothelioma cells	In vitro	MSTO-211H and H-2452 cells	30 µM	-	Increasing ROS generation Triggering loss of mitochondrial membrane potential Enhancing Bax/Bcl-2 ratio Apoptosis induction Providing CP sensitivity	[289]
Macrovipecetin	Melanoma	In vitro	SK-MEL-28 cells	0–1 µM	-	Impairing cancer proliferation Decreasing ROS levels, showing tumor-promoting role of ROS Promoting CP sensitivity	[290]
Indicaxanthin	Cervical cancer	In vitro	HeLa cells	60 µM	ROS/p53	Enhancing ROS levels Activating p53 and p21 Apoptosis induction	[291]
Hederagenin	Head and neck cancer	In vitro In vivo	AMC-HN2–10, SNU-1041, SNU-1066, and SNU-1076 cells	50 and 100 µM 100 and 200 mg/kg	Nrf2/ARE	Inhibiting Nrf2/ARE axis Enhancing p53 expression Subsequent increase in ROS levels Increasing GSH depletion Inducing cell death	[292]
Ethaselen	Leukemia	In vitro	K562 cells	1.5 µmol/L	TrxR/ROS	Increasing ROS generation via TrxR inhibition Bax upregulation and Bcl-2 down-regulation Cytochrome C release Apoptosis induction NF-κB down-regulation	[293]
Ascorbate	Osteosarcoma	In vitro	U2OS and 143B cells	0–100 µM	-	Increasing ROS levels to impair glycolysis and mitochondrial function in cancer cells Reducing cell sphere formation capacity Increasing CP sensitivity	[238]

## Data Availability

Data sharing not applicable.

## Abbreviations

CP: cisplatin; ER, endoplasmic reticulum; UPR, unfolded protein response; HAS, human serum albumin; P-gp, P-glycoprotein; GPX4, glutathione peroxidase 4; ROS, reactive oxygen species; NOX, NADPH oxidase; cyt C, cytochrome C; ERK, extracellular signal-regulated kinase; GRP78, glucose regulated protein 78; CHOP, C/EBP homologous protein; PTEN, phosphatase and tensin homolog; PCD, programmed cell death; JNK, c-Jun N-terminal kinase; CSCs, cancer stem cells; CAFs, cancer-associated fibroblasts; MDSC, myeloid-derived suppressor cell; HBV, hepatitis B virus; KLF4, krupple-like factor 4; miRNAs, microRNAs; STAT3, signal transducer and activator of transcription 3; TRIM32, tripartite motif 32; TKT, transketolase; SIRT2, sirtuin-2; IDH1, isocitrate dehydrogenase 1; PON, paraoxonase; Nrf2, nuclear factor erythroid 2-related factor 2; NSCLC, non-small cell lung cancer; PGC-1α, PPAR-gamma co-activator-1α; Nox2, NADPH oxidase 2; PI3K, phosphoinositide 3-kinase; Akt, protein kinase-B; GPX1, glutathione peroxidase 1; NF-κB, nuclear factor-kappaB; ABC, ATP-binding cassette; ABCB1, multidrug resistance protein 1; EFHD2, EF hand domain-containing protein 2; PKM2, pyruvate kinase isoenzyme type M2; PEP, phosphoenolpyruvate; TRX1, thioredoxin; TRAP1, tumor necrosis factor receptor-associated protein 1; elF2α, eukaryotic initiation factor 2α; PKR, protein kinase R; HRI, heme-regulated inhibitor; PERK, protein kinase R-like endoplasmic reticulum kinase; GCN2, general control nonderepressible-2; PRDX2, peroxiredoxin 2; HIF-1α, hypoxia inducible factor-1α; Drp1, dynamin-related protein 1; Mfn, mitofusin; PD-L1, programmed death ligand-1; DUOX1, dual oxidase 1; ATR, ataxia telangiectasia and Rad3-related protein; ChK1, Checkpoint kinase 1; SIRT5, sirtuin-5; HO-1, heme oxygenase-1; EMT, epithelial-to-mesenchymal transition; VEGFR, vascular endothelial growth factor receptor; CAM, clatrithromycin; MAPK, mitogen-activated protein kinase; XIAP, X-linked inhibitor of apoptosis protein; siRNA, small interfering RNA; shRNA, short-hairpin RNA; MSNs, mesoporous silica nanoparticles; mTOR, mammalian target of rapamycin; AMPK, AMP-activated protein kinase; LDH, lactate dehydrogenase.
